# A Little Experience with Leeches

**Published:** 1862-07

**Authors:** 


### A LITTLE EXPERIENCE WITH LEECHES.

When young in the profession, practicing in a country town, we often felt the want of leeches. Older physicians told us they would die before we were ready to use them, even if we obtained them; but if they didn't, they certainly would immediately after using them. Now, we are ready to tell young physicians and dentists that they need never be without these desirable pets; for they are much more easily cared for than canary birds or kittens, besides being less noisy than the former, and less filthy than the latter.

We have kept two of them, since last fall, in a pint jar with a perforated lid, by merely supplying them twice a week with fresh rain-water. They have been used a great many times, and appear as lively in twenty-four hours after use as before. When they have sucked themselves full of blood, we put them into a clean wash basin, sprinkle fine salt on them, and allow them to crawl and squirm till they disgorge, when we wash and return them to their home, with fresh rain-water.

Even the city dentist will find it less troublesome to keep them than to send for them when they are needed, while those in country towns must keep for themselves or do without.

We presume our brethren of the dental depots would furnish them to any of their customers when ordered. W.

